# Supplementary material for: Evaluation of factors associated with HIV self-testing Acceptability and Uptake among the MSM community in Nairobi, Kenya: A cross sectional study
Source: PLoS One. 2023 Mar 9;18(3):e0280540. doi: 10.1371/journal.pone.0280540 (PMC9997958; doi:10.1371/journal.pone.0280540)
Supplement: S1 Study tool — (DOCX) [file pone.0280540.s001.docx]

# 7.0 Annexures

## 7.1 Questionnaire

**Treatment as prevention among MSM in Nairobi & Neigbouring Counties**

**Respondents Characteristics**

I would like to ask you some general questions about yourself.

**100. Kindly indicate the following details below.**

| Your names first letter |  |
| --- | --- |
| Your mothers name first letter |  |
| Date of birth |  |
| District of Birth |  |

**101. What is your Age ? please indicate.................**

**102. Place of Birth (Country)............................**

**103 Place of Residence .................**

**104 What is your Religion?**

1􀀀Roman Catholic

2􀀀Protestant

3􀀀Muslim

4􀀀Traditional

5􀀀Hindu

6􀀀Buddhist

7􀀀Others

**105. Whats is your highest level of Educational Achievement ?**

1􀀀 Never

2􀀀 Primary School

3􀀀 Secondary School

4􀀀 Tertially education

**106.Has your sexual identity impacted on your educational attainment?**

1􀀀 Yes 2􀀀 No

􀀀 If yes, Kindly explain how.

.........................................

**107 Are you currently employed ?**

1􀀀 Yes 2 􀀀 No

a) If yes, *tick appropriately*

1􀀀 Full-time paid employment

2􀀀 Part-time paid employment

3􀀀 Self employed

4􀀀 Un-employed

b) Whats your monthly income.

1􀀀 Less than 6,000

2􀀀 6,000-25,000

3􀀀 25,001-75,000

4􀀀 More than 75,000

**108. Do you identify yourself as “men who have sex with men” (MSM)**

1􀀀 Yes 2􀀀 No

1. If yes how long have you been an MSM........................

**109 Do you identify yourself as” men sex worker” (MSW)**

**1**􀀀 Yes 2􀀀 No

**110 Do you identify yourself as Transgender?**

**1**􀀀 Yes 2􀀀 No

**111.What is your sexual orientation**

1􀀀 Homosexual

2􀀀 Bisexual

3􀀀 Heterosexual

4􀀀 others

**If others, kindly specify............**

**112. Marital status**

1􀀀 Single 4 􀀀 Divorced /Seperated.

2􀀀 Windower

3􀀀 Married

**If ever Married;**

1􀀀 Were you married to a man

2􀀀 Were you married to a woman

**113. Sexual attraction: How do you describe your feelings of sexual attraction at the moment?**

1􀀀 As a man who is attracted to men

2􀀀 As a man who is attracted to a woman

3􀀀 Attracted to both sexes

4􀀀 Unsure.

**114. How many sexual partners have you had sex with in the last six months?**

1􀀀 One 2􀀀Two 3􀀀Multiple 4􀀀 None

**115. Do you use condom during sex?**

**1**􀀀 Yes 2 􀀀 No

**If, yes how often**

1􀀀 Always 2􀀀 Most of the time 3􀀀 Sometimes 4􀀀 Never

**116. Do you prefer top or bottom?**

1􀀀 Top 2􀀀 Bottom 3􀀀 Both/Versatile

**117. Are you currently using the following?**

1􀀀 Alcohol 2􀀀 Hard drugs

**If yes, to hard drugs, kindly, indicate which ones?..................................**

**118. Have you ever had Anal sex after Alcohol/Hard drugs use ?**

**1**􀀀 Yes 2􀀀 No

**119. Do you use condoms during anal sex after alcohol/Hard drugs use?**

1􀀀 Always 2􀀀 Most of the time 3􀀀 Sometimes 4􀀀 Never

**120. Did you use a lubricant in your last sexual act?**

1 􀀀 Yes 2􀀀 No

1. **If yes, Kindly tick where appropriate**

1 􀀀 KY jelly
2 􀀀 Body cream
3 􀀀 Saliva
4 􀀀 Vaseline

**If others, kindly specify ……………..**

**121. Have you ever heard any mental health issues or disorder?**

1. 􀀀 Yes 2. 􀀀 No

**If yes, kindly explain…………….**

**122. Do you have an insurance cover?**

1􀀀 Yes 2􀀀 No

**123 Have you been tested for;**

**a) Testing**

|  | Infection | Ever in life | In last 6 months | Knows status, **-ve** | Knows status, **+ve** |
| --- | --- | --- | --- | --- | --- |
| i. | HIV/AIDS | ( ) | ( ) | ( ) | ( ) |
| ii. | Tuberculosis | ( ) | ( ) | ( ) | ( ) |
| iii. | Hepatitis C | ( ) | ( ) | ( ) | ( ) |
| iv. | STI | ( ) | ( ) | ( ) | ( ) |
| v. | Hepatitis B | ( ) | ( ) | ( ) | ( ) |

**b) Are you currently enrolled in any HIV Prevention Care & Treatment program.**

1􀀀 Yes 2􀀀 No

**If Yes, kindly specify ………………..**

**124. What social media pages do you frequent most? *Kindly select one***

1􀀀 Facebook 2 􀀀 Whats-up

3􀀀 Instagram 4 􀀀 Grinder

5 􀀀 Gay.com 6 􀀀 Badoo

7􀀀 Planet Romeo 8􀀀 Dating Buzz

9􀀀 Sex Trader

2. To explore determinates of HIV self test uptake among the MSM community

**125 (a) Have you ever tested for HIV/AIDS?**

1􀀀 Yes 2􀀀 No

(b) **If Yes, how often do you test for HIV/AIDS**

1􀀀Every 3 months 2􀀀Every 6 months 3􀀀Annually 4􀀀Greater than 1 year

**(c) If not tested before. What are the main reasons for not testing ? (*Kindly, select the***

***main three )***

1􀀀 I dont feel at risk

2􀀀 I am afraid of learning a postive HIV status

3􀀀 Time wasting

4􀀀 Service providers attitude

5􀀀 Lack of friendly services.

6􀀀 Due to stigma

7􀀀 Others specify..............................

**d) If yes, Where did you do the last test?**

1􀀀 Goverment facilities

2􀀀 Private hospital/ clinic

3􀀀 NGO

4􀀀 CBO and faith based facilities

5􀀀 Others, Kindly, specify..........................

**e) If yes, What was the reason for most recent testing? (*Kindly, select the***

***main reasons )***

1􀀀 Routine

2􀀀 Had unprotected anal sex

3􀀀  Had physical discomfort

4􀀀 Going to have a new partner

5􀀀 To confirm a previous result

6􀀀 Had sex under influence of alcohol

7 􀀀Other please specify………………..

**F) What was the result of the most recent HIV test?**

1􀀀 Negative

2􀀀 Positive

3􀀀 Undetermined

**126 Have you ever heard of window period?**

1􀀀 Yes  2 􀀀 No

a) **If yes did you considered the “window period” in deciding when to test ?**

1􀀀 Yes  2􀀀 No  3􀀀 Don’t know

**127. Would you consider taking up HIV self test as one of the HIV combination prevention strategy?**

1 􀀀 Yes 2 􀀀 No

**128. If you tested HIV positive through HIV self test, would you go for a confirmatory test after self test?**

1􀀀 Yes 2􀀀 No

**a) If yes how long would you take to go for confirmatory test?**

1􀀀 One week

2􀀀 Two weeks

3􀀀 Three weeks

4􀀀One Month

5􀀀 Would not go to confirm

**b). If the confimatory test shows negative, after how long would you seek for a re-test**

**after the current negative result test?**

1􀀀 0-3 months

2􀀀 4-6 months

3􀀀 7-12 months

4􀀀 Never re-test

**129. If you tested postive would you use protection or condoms with your partner?**

1􀀀 Yes 2􀀀 No

**a)Would you go for counselling after positive results?**

1􀀀 Yes 2􀀀 No

If yes where

1􀀀 Nearest Goverment Facility

2􀀀 Drop in centres

3􀀀 Private hospital

4􀀀 If any other specify......................................

b) **If you got positive results which healthcare provider would you want to approach first to seek support? (Kindly, select one)**

1􀀀 Health care provider

2􀀀 Partner

3􀀀 Peer Educator

4􀀀 Family Member

C)**. If you tested negative would you use condoms with your partner?**

1􀀀 Yes 2􀀀 No

**130. Have you ever heard of HIV Self test?**

1􀀀 Yes 2􀀀 No

**a) Have you heard of Oral HIV Self test?**

1􀀀 Yes 2􀀀 No

**b) Have you heard of Blood sample HIV Self test?**

1􀀀 Yes 2􀀀 No

**c) Have you used HIV test kit before?**

1􀀀 Yes 2􀀀 No

**131.What do you think of HIV Oral self test kit?**

1􀀀 Difficult to use

2􀀀 Easy to use

3􀀀 Not accurate

4􀀀 Dont know about it

**132.What do you think of HIV Blood self test kit?**

1􀀀 Difficult to use

2􀀀 Easy to use

3􀀀 Not accurate

4􀀀 Don’t know about it

**a) If you think self-test is difficult to use, who would you prefer to assit /guide you ?**

1􀀀 Health care provider

2􀀀 Partner

3􀀀 Peer Educator

4􀀀 Family Member

**b)If you are using self-test for the first time would you prefer to have a treament “buddy”**

**1􀀀 Yes 2. 􀀀 No**

c**).Would you prefer to use the Oral or Blood self test kits?**

1􀀀Oral Self test kit 2􀀀 Blood sample Self test kit

**c).What would prevent you from using the HIV oral test kit?**

1􀀀 Not available

2􀀀 Cost

3 􀀀 Lack of knoweldge

4􀀀 Fear

5􀀀Others specify...............................

**d).What would prevent you from using HIV Blood self test kit?**

1􀀀 Not available

2􀀀 Cost

3 􀀀 Lack of knoweldge

4􀀀 Fear

5􀀀Others specify................................

**e)** **Would you recommend your partner to use the self kit before sex?**

1􀀀 Yes 2􀀀 No

If no explain...............................................

**f). What do you think is the main advantage of self testing for HIV (Kindly, select one) ?**

1􀀀 Privacy/Anonimity/confidentaility

2􀀀 Personal empowernemnt /taking charge of ones health

3􀀀 No pricking/no pian

4􀀀 Saves time/no waiting in queues

5􀀀 I dont see any adavntage

6􀀀 Other specify...........................

**g) What do you think would be the main disadvantage of self testing for HIV (Kindly, select One)?**

1􀀀 Illetrate people may not be able to use the device

2􀀀 Difficulties in performing the test or interpleting results

3􀀀 Increased possibilty of self harm on a positive test

4􀀀 Testing others without consent

5􀀀 Production of fake or poor quality test

6􀀀 Reduced chances of enrolling in care

**133 Where would you want to pick the oral HIV self kit or what distribtion channels would you prefer? Kindly tick one.**

1􀀀 Retail or community pharmacy

2􀀀 Nearest Goverment Facility

3􀀀 NGO Facility/Drop in centres

4􀀀 Private hospital /Private clinic

5􀀀 Internet

6􀀀 Through Peer Educators

7􀀀 If any other specify......................................

**134. What do you think of the current cost (Approximately 700 Ksh)**

**of the HIV Self kit ?**

1􀀀 Expensive

2􀀀 Affordable

**135. If the test kit were to have a hotline, which institution or organisation would you prefer to provide the infomation?**

1􀀀 Public hospital

2􀀀 Private Hospital

3􀀀 Drop in centres

4􀀀 If any other specify.....................

**a) What are the reasons for choosing this facility?**

**1**􀀀 confidentality/Private

2􀀀 presence of skilled staff/Quality service

3􀀀 Easily accessible

4 􀀀 If any other specify.....................

**136 Would you prefer to visit the health facility for further support or you would prefer the healthcare provider to vist you at home for support, care & treatment?**

1􀀀 Visit Healthcare facility

2􀀀 Healthcare provider to make home visits

3􀀀 If any other specify.....................

**137. How often do you go for STI screening?**

1􀀀 0-3 months

2􀀀 4-6 months

3􀀀 7-12 months

4􀀀 Never

**138 Do you have any additional questions or Information?**

..................................................................................................................

***Thank you for your time***
