# Supplementary material for: Evaluation of factors associated with HIV self-testing Acceptability and Uptake among the MSM community in Nairobi, Kenya: A cross sectional study
Source: PLoS One. 2023 Mar 9;18(3):e0280540. doi: 10.1371/journal.pone.0280540 (PMC9997958; doi:10.1371/journal.pone.0280540)
Supplement: S1 Graph — (PDF) [file pone.0280540.s003.pdf]

Theoretical Framework

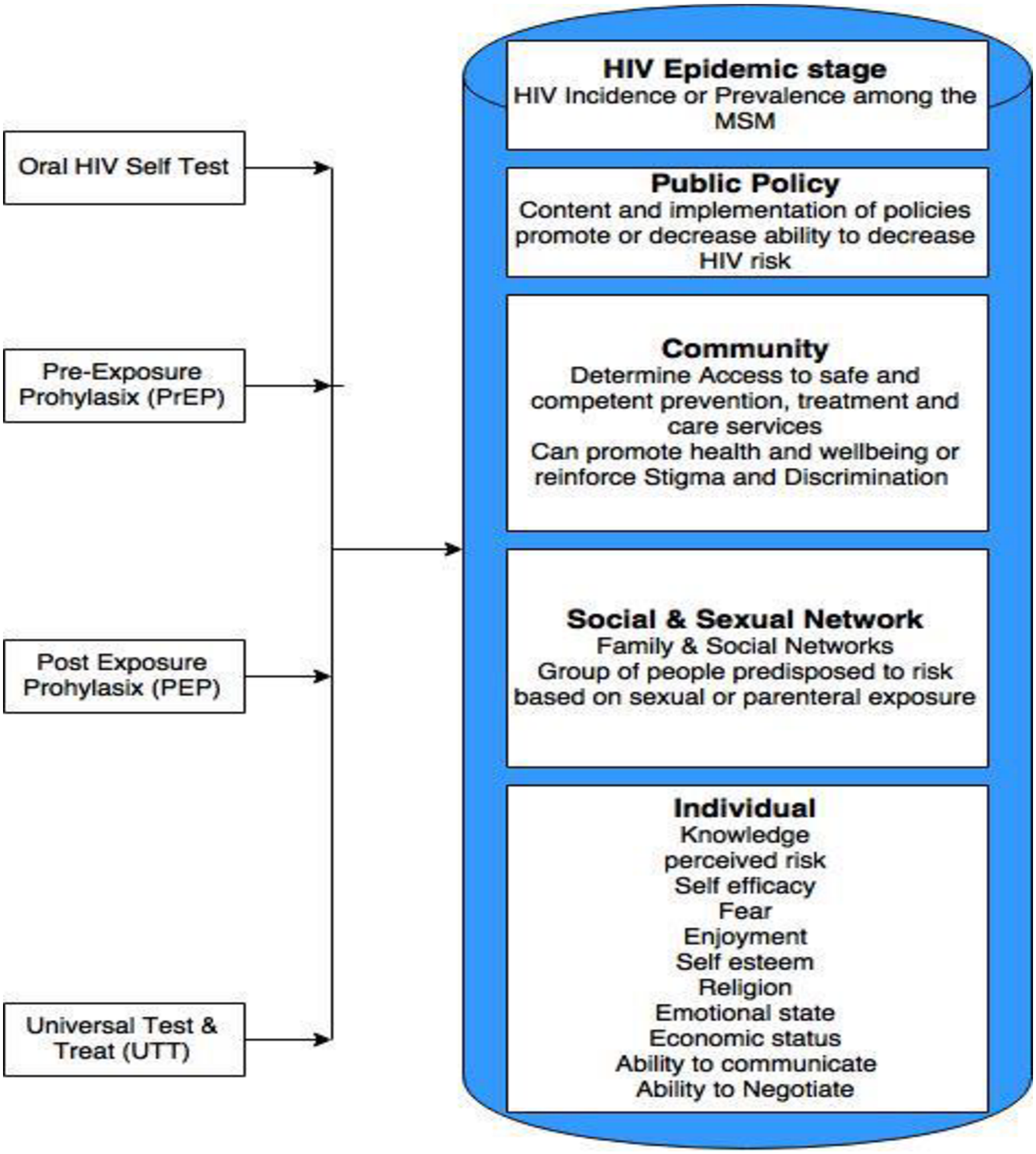

Figure 1: Theoretical Framework combining AIDS Risk Reduction Model (ARRM) and Modified Social Ecological Model showing the Interconnectedness of the different factors.
