# Supplementary material for: Evaluation of factors associated with HIV self-testing Acceptability and Uptake among the MSM community in Nairobi, Kenya: A cross sectional study
Source: PLoS One. 2023 Mar 9;18(3):e0280540. doi: 10.1371/journal.pone.0280540 (PMC9997958; doi:10.1371/journal.pone.0280540)

## Probability Plot of Normality tests

## Ever tested for HIV/AIDs

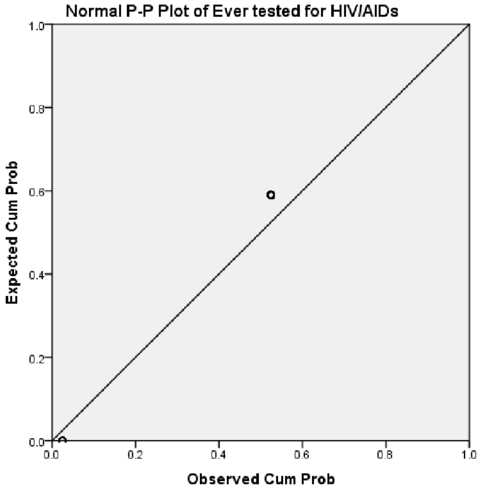


## Reason for most recent test

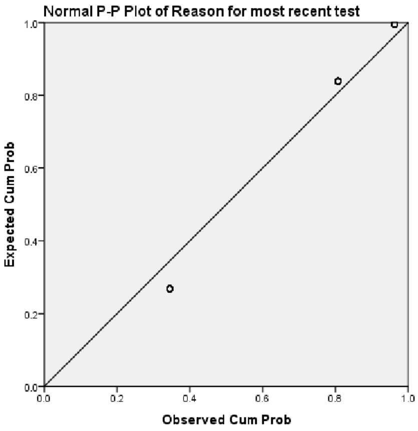


## If yes how often do you test for HIV/AIDS

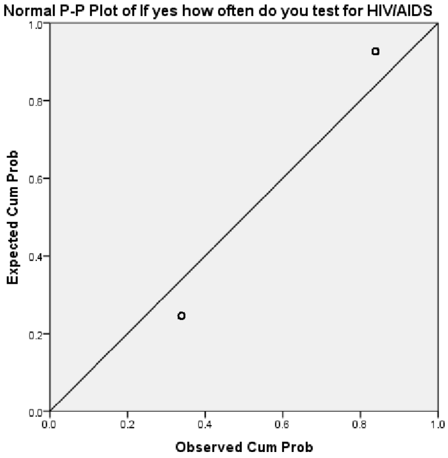


## Ever heard of window period

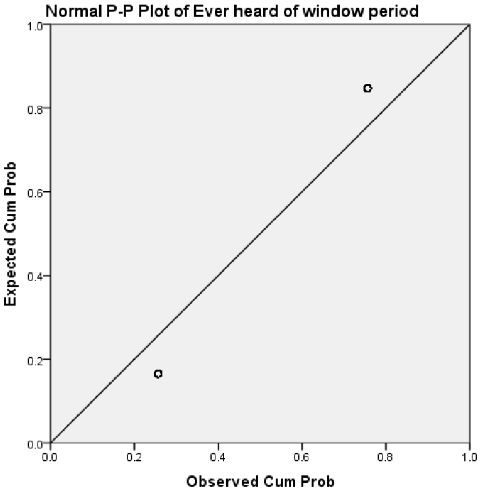

Supplement: S1 Plot — (PDF) [file pone.0280540.s004.pdf]
