# Supplementary material for: Evaluation of factors associated with HIV self-testing Acceptability and Uptake among the MSM community in Nairobi, Kenya: A cross sectional study
Source: PLoS One. 2023 Mar 9;18(3):e0280540. doi: 10.1371/journal.pone.0280540 (PMC9997958; doi:10.1371/journal.pone.0280540)
Supplement: Attachment — Submitted filename: HIVST Reviewers comments & Response July 29th.docx [file pone.0280540.s006.docx]

**Reviewer Comments & Response**

| **Reviewers Comments** | **Response** |
| --- | --- |
| **Reviewer #1** |  |
| Specifically, no information on bias or missing data although the response letter states this was done. | This comment is well received.  Statement on management of missing data on the questionnaires has been included in the abstract (line 41-43) and the methods (line 171-177). |
| As the primary outcome is based on self-report of past HIVST use this needs to be acknowledged at least in the limitations. | The study is based on respondent self-reports on HIVST use, and as such the veracity of such reports could not be easily determined within the design of this study. The above statement has been included in the discussion (Line 399-401) |
| The main outcome metric of HIV self-testing needs to be better described in the methods.  It seems that is based on participant report solely, which is fine but needs to be more clearly stated and how that also could suffer from bias | The operational definition of HIV self-testing has been in-cooperated in the methods (Line 192-197) |
| The updated draft with the regression model is difficult to follow on how it was constructed and run. Associated with that the reporting of the model is confusing as to what the main findings are and the tables have too much information on statical outputs (there is no need to report coefficients and the aPOR or the SE and the CIs). These aspects need to be corrected for publication. | The multivariate analysis has been explained how it was constructed and organized and was based on measure of association (Chi-Square) and risk ratio (POR) captured in the multivariate analysis (Line 284-285).  The table has also been reduced and only key outputs have been retained. Columns on S.E, Wald have been deleted |
|  |  |

.
