# Supplementary material for: Evaluation of factors associated with HIV self-testing Acceptability and Uptake among the MSM community in Nairobi, Kenya: A cross sectional study
Source: PLoS One. 2023 Mar 9;18(3):e0280540. doi: 10.1371/journal.pone.0280540 (PMC9997958; doi:10.1371/journal.pone.0280540)
Supplement: Attachment — Submitted filename: Response to Reviewers Dec 15th, 2022.docx [file pone.0280540.s007.docx]

PONE-D-21-07875R2

Evaluation of factors associated with HIV self-testing Acceptability and Uptake within the MSM community in Nairobi, Kenya: A cross sectional study.

**Reviewers & Authors Comments.**

Good progress by the author team. This is an improved draft.

| **Reviewers Comments** | **Response** |
| --- | --- |
| Journal Requirements:  Please review your reference list to ensure that it is complete and correct. If you have cited papers that have been retracted, please include the rationale for doing so in the manuscript text, or remove these references and replace them with relevant current references. Any changes to the reference list should be mentioned in the rebuttal letter that accompanies your revised manuscript. If you need to cite a retracted article, indicate the article’s retracted status in the References list and also include a citation and full reference for the retraction notice. | The references have been carefully checked to ensure they are complete and non-has been retracted. |
|  |  |
| Reviewer #1  The authors state the missing data were MCAR to justify the listwise deletion. Please provide the information on how this was assessed and add that to the methods and/or results as appropriate. If a graphical method was utilized this can be provided as a supplement for the readers. | Graphical method was utilized to inform the adoption of listwise deletion. Graphs have been attached in the appendix |
| The tables are more understandable in this format. For the a POR table please indicate (perhaps footnotes) what the reference categories are. All tables and figures should be interpretable by themselves. | The comment is well received. All the tables have been reformatted to be self -explanatory We have Introduced borders and the Ref. to distinguish the reference variables. |
|  |  |
